# Arterial Stiffness and Age Moderate the Association Between Physical Activity and Cognition in Older Adults

**DOI:** 10.1093/geroni/igab046.207

**Published:** 2021-12-17

**Authors:** Adrian Noriega de la Colina, Atef Badji, Maxime Lamarre-Cliche, Louis Bherer, Hélène Girouard, Navin Kaushal

**Affiliations:** 1 McGill University, Montreal, Quebec, Canada; 2 Université de Montréal, Montreal, Quebec, Canada; 3 University of Indiana, Bloomington, Indiana, United States

## Abstract

Background: Evidence supports that time spent on physical activity has beneficial effects on cognition in older adults. Nevertheless, this beneficial effect is likely to change in function of individual modifying factors like age and level of arterial stiffness. This study aims to reveal whether arterial stiffness and age modulate the positive impact of physical activity on cognition by developing a double moderation model. Methods: 110 healthy older adults aged 60 to 75 years old were examined for arterial stiffness (carotid-femoral Pulse Wave Velocity [cf-PWV]), global cognition (composite score of Montreal Cognitive Assessment, and Mini-Mental State Examination), and self-reported physical activity (PACED diary). Using PROCESS macro for SPSS, we evaluated if cf-PWV (moderator 1), and age (moderator 2) moderate the relationship between physical activity (X) and global cognition (Y). The threshold for high stiffness was set at 8.5 m/s based on previous studies that reported this cut-off more appropriate for classifying cerebrovascular risk groups. Results: The interaction of arterial stiffness x age moderated the effect of physical activity on global cognition (β=-.89, SE=.42, p=.037) (Model: R2=.15, p=.018). Physical activity had a positive effect on cognition in younger-older adults (aged 60 to 68.5 years) with cf-PWV>8.5 m/s (β=.57, SE=.222, p=.011, 95% CI.133 to 1.014) and on older-older adults (aged 68.6 to 75 years) with cf-PWV<8.5 m/s (β=.49, SE=.190, p=.010, 95% CI=.116 to .869). Conclusions: Identifying the right age groups and arterial stiffness levels at which physical activity can have beneficial effects on cognition is a key step in providing tailored behavioral interventions.

